# Reflections on the Addis Ababa Immunization Conference of 2016

**DOI:** 10.11604/pamj.2016.23.63.9220

**Published:** 2016-02-29

**Authors:** Robert Davis

**Affiliations:** 1 American Red Cross, Nairobi, Kenya

**Keywords:** Immunization, Africa, Expanded Program on Immunization, African Union

## Perspective


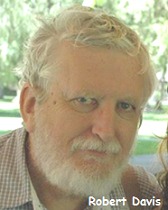


The inter-ministerial conference on immunization held at African Union headquarters in February 2016 came, by coincidence, almost exactly 30 years after the first international immunization conference I attended, convened by World Health Organization (WHO) and UNICEF in Mbabane in December 1985. Times have changed. In 1985, there was no Gates Foundation, there was no Global Vaccine Alliance (GAVI Alliance), and there was no global commitment to polio eradication. Three items which figured prominently on the Addis Ababa program were quite absent from many earlier meetings: 1) the economic justification for vaccination and eradication; 2) the role of parliamentarians, religious leaders and civil society organizations (CSOs) in advocacy and accountability for vaccination programs, 3) vaccine financing.

## The economic justification for vaccination and eradication

Economists were not much to be seen in the vaccination programs of 30 years ago. Vaccination, like virtue, was thought to be its own reward. This assumption was based on the erroneous premise that finance ministers would support vaccination programs because they were a global public good, like clean air. The multiple calls on scarce public resources have generated much academic work to build the case for vaccination. The most quoted figure from the Addis Ababa conference was based on the published work of Ozawa and colleagues, from Johns Hopkins, showing a benefit: cost ratio of 16:1 for childhood vaccinations in low- and middle-income countries. Their work [1] is a powerful advocacy tool for use with hard-nosed finance ministers, who must say no to health ministries as often as they say yes.

The conference coincided with the publication of a newspaper interview by Wycliffe Muga with Dr. Kimberly Thompson on the subject of disease eradication [2].

## Parliamentarians, CSOs and religious leaders

Thirty years ago, vaccination was a program to be managed by national health ministries with the support of international agencies, especially W.H.O. and UNICEF. That model worked poorly because it assumed that governments always act in the interest of their citizens. When governments cut corners, it is often at the expense of their most vulnerable citizens, especially children.

Civil society organizations and parliamentarians, formerly marginal players in immunization, have assumed growing importance in assuring that governments adhere to their commitments properly to finance and support health services in general, and immunization in particular.

This need is all the more pressing as vaccines used and vaccine procurement bills have seen an astronomical rise. Under these conditions, it is not surprising that 9 African countries are in default on their co-payments for internationally procured vaccines. When this information becomes widely known, pressure builds on governments to take corrective measures.

One parliamentarian from Kinshasa illustrated the point during the Addis Ababa conference.

“When the Prime Minister says the co-financing is done, I phone UNICEF. If UNICEF does not confirm, I phone him again.”

The role of religious leaders in vaccination became clear during the Nigerian vaccination boycott of 2003, which slowed the elimination of polio from that country, only polio free since 2014. The Sultan of Sokoto, speaking at the Addis Ababa conference, serves as chair of the Council of Traditional Rulers. Speaking in Addis Ababa, he said the following about the vaccine boycott.

“Nigeria had a role in the fight against polio, especially the OPV boycott, until the traditional leaders intervened in favor of the polio campaigns. The people of Nigeria give a lot of respect to their religious leaders. Our intervention is on the positive side. Our common enemy was polio. With the Federal Ministry of Health, I worked on this issue. When I say “religious leaders,” I mean both Muslims and Christians. When you work together, setting aside your divisions, you can solve the problem and make the lives of Nigerians much better.”

Among most Christians, there has been general support for vaccination, as illustrated by Pope Francis’ recent participation in Mexico's success polio vaccination project (see photo). Where opposition has appeared, as on the part of the Kenyan Episcopal Conference, it has been generally sporadic. Health education among Catholic and non-Catholic communities has in the case of Kenya blunted the edge of the bishops’ campaign (unpublished data, Dr. Collins Tabu, Kenyan Ministry of Health).

In the 21st as in the 20th century, health services provided by faith based organizations have been a point of entry for vaccination and other child health services, sometimes providing vaccination services in places where government health services have broken down. In the Zaïre of Mobutu, the largest vaccine cold chain in Mayumbe was run from the mission hospital in Kangu. With reliable standby generators, it was the only hospital in Mayumbe with reliable power supply 24/7 during the late ‘70s.

Taken as a whole, the religious participation in vaccination is a decided plus.

## Vaccine Financing

The world's vaccine bill is on the rise, as countries which used to vaccinate against six diseases now vaccinate against 10 to 12 diseases with vaccines which, at as much $5 a dose, are far less affordable than in the past. Thirty years ago, the Republic of Kenya had a vaccine bill for its routine vaccines (BCG, measles, DPT, OPV and TT) of < $1 million. Today, Kenya's bill for 11 vaccines exceeds $20 million, most of it for new and under-used vaccines co-financed by the Kenyan government and international partners.

The cost of vaccines is especially tragic in the fight against cancer. Hep B vaccine, available from multiple suppliers at low prices, is now given in all African countries. Impact studies have shown significant declines in Hep B prevalence among early adopters of Hep B vaccine, and liver cancer is likely to show similar declines in future decades. For HPV, human papillomavirus vaccine, Rwanda, in sub-Saharan Africa, has introduced this vaccine on a national scale, and that with full external funding. Most other African countries have introduced HPV on a sub-national scale, or not at all. Meanwhile, cervical cancer remains a major cause of death among women of child bearing age.

The international community has increased its spending on vaccines, as have developing countries. But gaps remain. Donal Brown, speaking for one major donor (DFID), made the following comments from a partner perspective.

“There is enormous competition for donor resources. Everything is a priority, but you can't fund anything. Spending on humanitarian relief last year was the highest in history. EPI competes with other priorities.

The Johns Hopkins benefit: cost figure of 16:1 comes to 44:1 when you incorporate all benefits. Immunization is the best buy. It is, therefore, something that developing countries should be willing to finance. We've looked at costs of immunization as a part of government expenditure. Most countries can pay for vaccination. It is a question of choice. There is a role here for community service organizations (CSOs). Revenues of African governments have almost tripled in the last decade. Donors cannot fill that gap. GAVI graduation policy (for countries with per capita GNI greater than USD 1500) is well considered.

It's a question of willingness to pay, not ability to pay. We need better engagement between finance ministries and health ministries. It's not a donor issue.”

Pooled procurement of vaccines, as in the Region of the Americas, was discussed by some participants in Addis Ababa. Another possible solution raised was the production of WHO prequalified vaccines in Africa. This is already done, by the Pasteur Institute of Dakar, for yellow fever vaccine. Chris Elias, of the Gates Foundation, suggested the following considerations on local vaccine production in Africa: 1) Regulatory environment. The regulatory hurdles are high for vaccines. Even successful companies stumble at times, sometimes losing their W.H.O. prequalification status, 2) India and China have had a long path. They had a large domestic population as their first market. Senegal has long been a prequalified supplier of yellow fever vaccine. Here in Ethiopia, The Bill and Melinda Gates Foundation (BMGF) has been working on production of veterinary vaccines, costing $50 million. The regulatory challenge is lower for veterinary than for human vaccines.

## Future Perspectives

With polio eradication in perspective, African countries are beginning to look for new targets for elimination, including measles and, perhaps, rubella. What are the preconditions to be met? The ideas emerging from the Addis Ababa conference pointed to the following imperatives:

- National political and economic commitments from health and finance ministries
- Uninterrupted financing of vaccine and local currency costs of vaccination from national and international partners
- Full participation in vaccination by religious leaders, CSOs, parliamentarians, and opinion leaders.

## References

[1] http://www.childsurvival.net/?content=com_articles&artid=5562.

[2] http://www.kidrisk.org/.

